# Effects of Autoclave Sterilization and Multiple Use on Implant Scanbody Deformation In Vitro

**DOI:** 10.3390/ma15217717

**Published:** 2022-11-02

**Authors:** Takamitsu Kato, Noriyuki Yasunami, Akihiro Furuhashi, Koma Sanda, Yasunori Ayukawa

**Affiliations:** Section of Implant & Rehabilitative Dentistry, Division of Oral Rehabilitation, Faculty of Dental Science, Kyushu University, Fukuoka 812-8582, Japan

**Keywords:** dental implants, accuracy, scanner, dental implant abutment, sterilization, deformation

## Abstract

In the intraoral scanner (IOS) impression technique for dental implants, a scanbody (SB) is connected to the implant and scanned. Poly(ether-ether-ketone) (PEEK) is a widely used material for SBs and it is recommended for single use. However, from the perspective of the Sustainable Development Goals, it is desirable to use these products multiple times. As SBs are used in patients’ mouths, proper sterilization is necessary for multiple uses. In the present study, the effect of autoclave treatment and connection/disconnection on SB deformation was investigated. The SB was connected to the implant and stereolithography (STL) data were obtained. Then, the SB was disconnected and underwent autoclave treatment, or was connected and disconnected multiple times, or underwent a combination of both processes. The results showed that there were significant differences in the distance and angle when comparing SBs before and after the autoclave treatment, but repeated connections with or without autoclave treatment had no significant impact on the measured values. The surface texture, observed with scanning electron microscopy, showed that a groove was observed on the surface of the SB, but the groove did not show major changes after 10 connection/autoclave processes. These results indicate that autoclave sterilization has some impact on SB deformation but connection/disconnection itself may not have a huge impact on SB deformation.

## 1. Introduction

With the development of digital technology, many new concepts have been introduced into the dental field. The use of an intraoral scanner (IOS) is one of the main parts of digital dentistry. As dental implants are industrially fabricated products, they are closely linked with digital technology, as compared to hand-prepared abutments or mucosa.

For the long-term stability of a dental implant, the accuracy of the superstructure of each dental implant is one important factor. In order to fabricate an accurate superstructure, the making of an accurate impression and the creation of a subsequent model are one of the important procedures [1,2,3,4,5,6,7]. In conventional dental implant treatments, impression copings and silicone materials, as well as sometimes the metal jigs and acrylic resin, are used. In the IOS technique, using a scanning scanbody (SB), the three-dimensional locations of individual implants are transferred to the software, together with the stereolithography (STL) data for the neighboring teeth and mucosa. Some studies have been conducted regarding the accuracy of the IOS technique and superstructures fabricated with digital technology, some of which indicate greater accuracy in the IOS technique compared to the conventional technique [8,9,10].

Scanbodies have a scan region on the upper part. By scanning this region, data on the implant position and the type of the implant are obtained. Various materials can be used for SBs, namely, titanium, a combination of titanium and poly(ether-ether-ketone) (PEEK), and PEEK only. The merit of titanium SBs is that titanium is strong material, it can be used many times, and it is autoclavable. However, as it is metal, it reflects light, which makes it difficult to scan intraorally [11]. SBs made with a combination of titanium and PEEK, in which the part connected to the implant body is made of titanium, are strong enough for multiple uses. Furthermore, in these SBs, the scan region is made of PEEK, which does not reflect light, which makes it easy to scan. However, the connection of the titanium base and the PEEK superstructure may lead to variations among these products. SBs made of PEEK alone have been reported to have more accuracy when scanned, compared with the other two types of SBs [11].

PEEK is a polyaromatic semi-crystalline thermoplastic polymer with sufficient mechanical properties for bio-medical applications. As its Young’s modulus and tensile properties are close to those of human bones and teeth, PEEK has been utilized for prosthodontic materials [12,13,14,15,16].

In order to prevent any potential deformation, SBs made of PEEK are recommended for single use. However, the accuracy of scan data obtained with SBs made of PEEK have been reported not to show significant differences after being hand-tightened up to 10 times [17], although the exact torque was not clarified in that report. As it has been reported that the torque of hand-tightening ranges from 11 Ncm to 38 Ncm [18], the tightening torque has not been standardized. Furthermore, as PEEK is a plastic material, deformation after autoclave treatment may occur.

In the dental field, any equipment used in a patient’s mouth is recommended to undergo proper sterilization before being used on another patient. There are many sterilization methods, such as autoclave sterilization, dry-heat sterilization, and ethylene oxide gas (EOG) sterilization. Furthermore, there are many disinfection methods, such as boiling, steam exposure, ultraviolet irradiation, disinfectant immersion [19,20], and so on. As PEEK scan bodies are recommended for a single use, no reports have been published regarding their sterilization.

Autoclave sterilization is commonly used in the dental field as it offers a high level of sterilization. Therefore, in the present study, the effect of autoclave treatment on PEEK SB deformation was evaluated.

In the medical field, disposable products are commonly used. Sometimes, this is not only because of the need for aseptic treatment, but because the cost in terms of human resources to clean the device is greater than the cost of using disposable products. However, for a sustainable society, developing a system that enables reuse whenever possible is essential. In that context, it is important to clarify the possibility of the multiple use of PEEK SBs.

The purpose of this study was to evaluate the effect of repeated torquing, sterilization and combination of both on the accuracy of PEEK tissue and bone level SBs.

## 2. Materials and Methods

**Figure 1:**
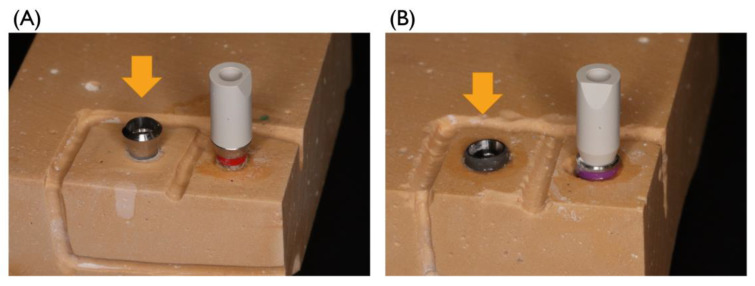
An implant analog for TL (**A**) or BL (**B**) on the right and an implant body for TL on the left were placed in improved dental stone, and the SB was tightened onto the implant analog on the right as a reference. Then, an SB was tightened on the left implant body (arrows) with a standardized torque (15 Ncm) and the distance and angle between two scan bodies were measured.

The experiments were performed according to a previous report [21], with slight modifications. Two types of models were fabricated—one was a tissue-level implant (TL) (Figure 1A) and the other was bone-level implant (BL) (Figure 1B). For TL, a Straumann Standard Plus Regular Neck (RN) φ 4.1 × 10 mm implant body (Straumann, Basel, Switzerland) and an RN SynOcta implant analog (Straumann) were placed in improved dental stone (New Fujirock Improved Dental Stone Golden Brown, GC, Tokyo, Japan). For BL, a Straumann BL Regular CrossFit (RC) φ 4.1 × 10 mm (Straumann) and an RC implant analog (Straumann) were placed in an improved dental stone (GC). The distance between the implant body and analog was adjusted to 11 mm. For SBs, CARES Mono SB RN (Straumann) for TL and CARES Mono SB RC (Straumann) for BL were connected to the implant body and the analog. A digital torque wrench (Newton–1, Kyoto Tool, Kyoto, Japan) was used to tighten the screw with 15 Ncm.

**Figure 2:**
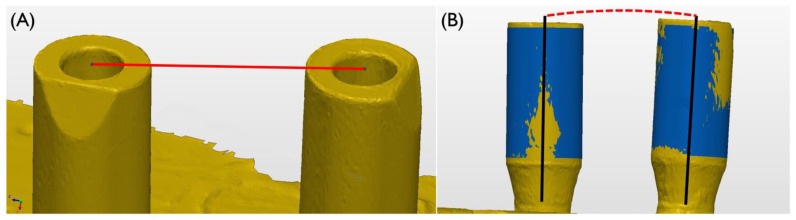
(**A**) The distance between the centers of the top surfaces of the two SBs was measured. (**B**) The angle between the axes of the two SBs was measured.

SBs were scanned using an IOS (TRIOS3, version 21.4.2, 3Shape, Copenhagen, Denmark). Acquired STL data were imported to the morphology measuring software (PolyWorks Inspector, version 18.9.6181, InnovMetric software, Québec, QC, Canada). The distance between the center of the upper surface of the SBs and the angle between the two SBs were measured and compared (Figure 2).

### 2.1. The Effect of Autoclave Treatment

For experiments 1 and 2, the effect of autoclave treatment on the deformation of the PEEK SBs was measured. Experiment 1 was carried out with TL, and experiment 2 was carried out with BL. New SBs were connected to the implant body or analog and STL data were acquired. These data were used as baseline data. Then, the SBs were disconnected and underwent autoclave treatment. Autoclave treatment was performed at 135 °C for 3 min (YS-A-C107J, YUYAMA, Osaka, Japan). After the autoclave treatment, the SBs were connected to the implant body or analog and STL data were acquired. For the control, the disconnected SBs were connected again and the STL data were acquired. For the experimental group and control group, the distances and angles calculated from the STL data were compared with the baseline data. Five SBs were used in experiments 1 and 2, respectively.

### 2.2. The Effect of Repeated Tightening on the Deformation of the Scanbodies

For experiments 3 and 4, the effect of repeated connection and disconnection on the deformation of the SBs was evaluated. Experiment 3 was carried out with TL and experiment 4 was carried out with BL. New SBs were connected to the implant and the analog and the STL data were obtained, which were used as the baseline. Then, the SBs were disconnected and connected with a standardized torque (15 Ncm). The STL data were obtained and compared to the baseline. The connections and disconnections were repeated 10 times. The difference between the baseline and the data obtained for each STL were compared. Five SBs were used in experiments 3 and 4, respectively.

### 2.3. The Combined Effect of Autoclave Treatment and Repeated Tightening on Deformation of Scanbodies

For experiment 5 and 6, the effect of autoclave treatment and repeated connection and disconnection on the deformation of the SBs was evaluated. Experiment 5 was carried out with TL and experiment 6 was carried out with BL. The new SBs were connected to the implant and analog and STL data were acquired for the baseline. Then, the SBs were disconnected and underwent autoclave treatment. Then, the SBs were connected and scanned. The STL data were compared with the baseline. This procedure was repeated 10 times. The differences between the baseline and the data for each STL were compared. Five SBs were used in experiments 5 and 6, respectively.

### 2.4. Surface Texture of the Connecting Area of the Scanbodies

For experiment 7, the surface texture of the connecting area of the BL SB was observed using scanning electron microscopy (SEM) (S-3400N, Hitachi, Tokyo, Japan) with magnification of 20× and 100× before and after each experiment. Samples were mounted on stubs, coated with an Au/Pd alloy, and evaluated microscopically.

### 2.5. Statistical Analysis

Student’s *t*-test was used for experiment 1 and 2 after the confirmation of the consistency of the results with a normal distribution by means of the Kolmogorov–Smirnov test. The Tukey test was used for experiments 3, 4, 5, and 6 after the confirmation of the results’ consistency with a normal distribution by means of the Kolmogorov–Smirnov test and the Bartlett test. *p*-values less than 0.05 were considered significantly significant. R (version 3.6.3, The R Foundation, Vienna, Austria) was used for all statistical analyses [22].

## 3. Results

### 3.1. The Effect of Autoclave Treatment on the Deformation of the Scanbodies

**Figure 3:**
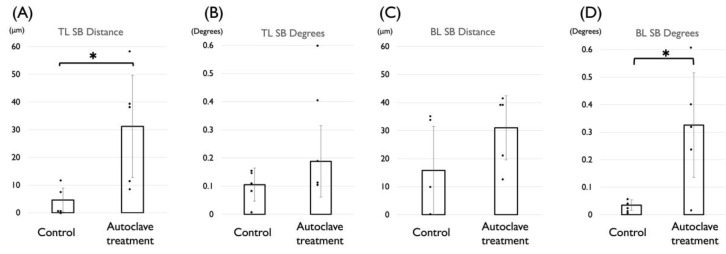
The effect of autoclave treatment on the distance and angle between the scanbodies. Student’s *t*–test, *: *p* < 0.05. (**A**) Distance of TL SB. (**B**) Angle (in degrees) of TL SB. (**C**) Distance of BL SB. (**D**) Angle (in degrees) of BL SB.

Based on experiment 1, the effect of autoclave treatment on the deformation of the TL SB was measured. The mean distance for the control group were 4.6 μm (SD 4.4) ranging from 0 to 12.0 μm, respectively. The mean distance and the standard deviation for the group that had undergone autoclave treatment were 31.2 μm (SD 18.4) ranging from 9.0 to 58.0 μm, respectively. This was a statistically significant difference (Figure 3A). The mean angle and the standard deviation for the control group were 0.11 degrees (SD 0.06 ranging from 0.01 to 0.17 degrees, respectively. The mean angle and the standard deviation the group that had undergone autoclave treatment were 0.19 degrees (SD 0.13) ranging from 0.06 to 0.43 degrees, respectively. There was no significant difference between the two groups (Figure 3B).

Based on experiment 2, the effect of autoclave on the deformation of the BL SB was measured. The mean distance and the standard deviation for the control group were 15.8 μm (SD 15.7) ranging from 0 to 35.0 μm, respectively. The mean distance and the standard deviation for the group that had undergone autoclave treatment were 31.0 μm (SD 11.4) ranging from 13.0 to 42.0 μm, respectively. This result was not significantly different (Figure 3C). The mean angle and the standard deviation for the control group were 0.04 degrees (SD 0.02) ranging from 0.02 to 0.06 degrees, respectively. The mean angle and the standard deviation for the group that had undergone autoclave treatment were 0.33 degrees (SD 0.19) ranging from 0.04 to 0.62 degrees, respectively. There was a significant difference between the two groups (Figure 3D).

### 3.2. The Effect of Repeated Tightening on the Deformation of the Scanbodies

**Figure 4:**
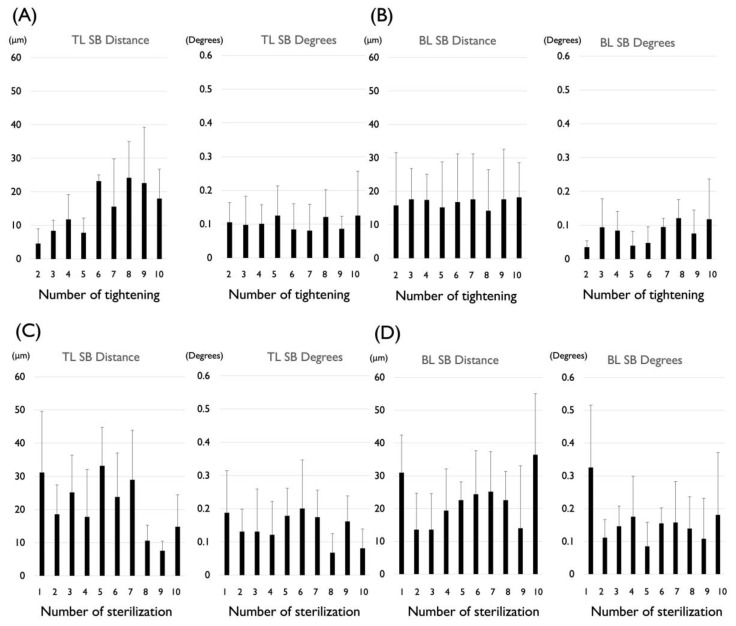
(**A**) The effect of repeated connection and disconnection on the deformation of the TL SBs. (**B**) The effect of repeated connection and disconnection on the deformation of the BL SBs. (**C**) The effect of autoclave treatment and repeated connection and disconnection on the deformation of the TL SBs. (**D**) The effect of autoclave treatment and repeated connection and disconnection on the deformation of the BL SBs.

Based on experiment 3, the effect of repeated connection and disconnection on the deformation of the TL SBs was evaluated. The largest mean distance and standard deviation after the disconnecting and connecting ten times were 24.2 μm (SD 10.7) ranging from 13.0 to 39.0 μm, respectively. The smallest mean distance and the standard deviation for the group that had undergone disconnecting and connecting 10 times were 4.6 μm (SD 4.4) ranging from 0 to 12.0 μm, respectively. This was not significantly different. The largest mean angle and the standard deviation for the group that had undergone disconnecting and connecting 10 times were 0.13 degrees (SD 0.09) ranging from 0.02 to 0.26 degrees. The smallest mean angle and the standard deviation for the group that had undergone disconnecting and connecting 10 times were 0.08 degrees (SD 0.08) ranging from 0 to 0.23 degrees, respectively. There was no significant difference (Figure 4A).

Based on experiment 4, the effect of repeated connection and disconnection on the deformation of the BL SBs was evaluated. The largest mean distance and the standard deviation for the group that had undergone disconnecting and connecting 10 times were 18.2 μm (SD 10.3) ranging from 0 to 26.0 μm. The smallest mean distance and the standard deviation for the group that had undergone disconnecting and connecting 10 times were 14.2 μm (SD 12.3) ranging from 5.0 to 38.0 μm, respectively. This was not significantly different. The largest mean angle and the standard deviation for the group that had undergone disconnecting and connecting 10 times were 0.12 degrees (SD 0.06) ranging from 0.03 to 0.19 degrees. The smallest mean angle and the standard deviation for the group that had undergone disconnecting and connecting 10 times were 0.04 degrees (SD 0.02) ranging from 0.01 to 0.06 degrees, respectively. There was no significant difference in these results (Figure 4B).

### 3.3. The Combined Effect of Autoclave Treatment and Repeated Tightening on the Deformation of Scanbodies

Based on experiment 5, the effect of autoclave treatment and repeated connection and disconnection on the deformation of the TL SBs was evaluated. The largest mean distance and the standard deviation for the group that had undergone autoclave treatment and disconnecting and connecting 10 times were 33.2 μm (SD 11.5) ranging from 18.0 to 50.0 μm. The smallest mean distance and the standard deviation for the group that had undergone autoclave treatment and disconnecting and connecting 10 times were 7.6 μm (SD 2.9) ranging from 2.0 to 10.0 μm, respectively. These results were not significantly different. The largest mean angle and the standard deviation for the group that had undergone autoclave treatment and disconnecting and connecting 10 times were 0.20 degrees (SD 0.15) ranging from 0.01 to 0.44 degrees. The smallest mean angle and the standard deviation for the group that had undergone autoclave treatment and disconnecting and connecting 10 times were 0.07 degrees (SD 0.06) ranging from 0 to 0.16 degrees, respectively. There was no significant difference in these results (Figure 4C).

Based on experiment 6, the effect of autoclave and repeated connection and disconnection on the deformation of the BL SBs was evaluated. The largest mean distance and the standard deviation for the group that had undergone autoclave treatment and disconnecting and connecting 10 times were 36.4 μm (SD 18.6) ranging from 10.0 to 63.0 μm. The smallest mean distance and the standard deviation for the group that had undergone autoclave treatment and disconnecting and connecting 10 times were 13.6 μm (SD 11.0) ranging from 4.0 to 28.0 μm, respectively. These results were not significantly different. The largest mean angle and the standard deviation for the group that had undergone autoclave treatment and disconnecting and connecting 10 times were 0.33 degrees (SD 0.19) ranging from 0.04 to 0.62 degrees. The smallest mean angle and the standard deviation for the group that had undergone autoclave treatment and disconnecting and connecting 10 times were 0.08 degrees (SD 0.07) range from 0.01 to 0.22 degrees, respectively. There were no significant differences in these results (Figure 4D).

### 3.4. Surface Texture of the Connecting Area of the Scanbodies

**Figure 5:**
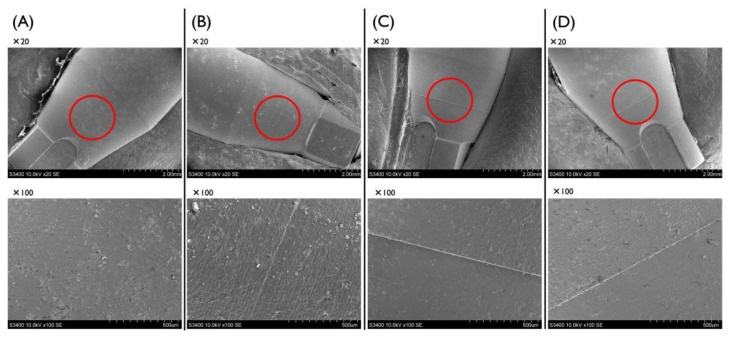
SEM observations of the surface of BL SBs. (**A**) The surface of an unused BL SB (20× and 100×). (**B**) The surface of a BL SB that had been connected once (20× and 100×). (**C**) The surface of a BL SB that had been connected/disconnected ten times (20× and 100×). (**D**) The surface of a BL SB that had received both autoclaving treatment and connection/disconnection ten times (20× and 100×).

In experiment 7, the surface texture of the connecting area of the BL SB was observed using scanning electron microscopy. No groove was observed on the surface of the unused BL SB (Figure 5A), but a groove was observed on the surface of the BL SB that had been connected once (Figure 5B). There were grooves on the surfaces of BL SBs that had been connected and disconnected 10 times and in the combination of multiple connections and autoclave treatment (Figure 5B–D). Comparing the group that had undergone connection and disconnection 10 times and the group that had undergone the combination of autoclave treatment and connecting and disconnecting 10 times, no apparent differences were observed.

## 4. Discussion

The data from experiments 1 and 2 indicated that the autoclave treatment had some impact on the measured values. It has been reported that PEEK medical devices showed up to 6% decreases in their lateral dimensions with specific shaped experimental clips after 50 cycles of sterilization at 121 °C for 30 min [23]. This report supports our findings obtained at 135 °C. As the glass-transition temperature of PEEK is 143 °C, slight deformation may start around the temperature used in the present study [24].

The connection between the SB and the implant body in the TL system was not identical to that in the BL system. Specifically, the SB used for TL was set “on” the implant body, but the SB for BL was set “in” the implant body. The former SB was set on the top of implant body. Thus, a vertical position is supposed to be relatively stable under the influence of the axial force generated by the tightening of a screw because a counterforce against the axial force is generated at the surface of the top of the implant, perpendicularly to the fixation screw. The latter SB type is inserted into the implant body and friction between the inserted SB and the inner surface of the implant body acts as a counterforce against the axial force. This counterforce is generated almost parallel to the tightening of the screw and if the screw is tightened strongly the SB may sink more than expected. This implies that the precision of the surface of the insert part of the SB strongly influences the position of the SB. As a result, it can be hypothesized that the precision of the measured value for TL is higher than that for BL. In the present study, we did not compare the precision in the distance and angle data of TL and those of BL because we placed the implant analog and implant body into dental stones and it was impossible to place TL perfectly at the same angle and distance compared with that of BL. Future testing of this hypothesis may provide further suggestions regarding the selection of the implant body.

The data from experiments 3 and 4 indicated that the repeated tightening of the SBs under the standardized torque of 15 Ncm did not cause an apparent deformation of the SBs. In a previous study, reusing SBs up to 10 times did not seem to consistently affect the accuracy of digital impressions [17]. This is consistent with the findings of the present study. Thus, we can suggest that repeated tightening itself will not cause significant deformation.

In the experiment 5 and 6, significant deference was not detected. This may be because deformation after autoclave treatment and following tightening interfere each other so that the measured data was contaminated. It can be speculated that the deformation of autoclave treatment and tightening would occur in different dimensions.

In experiment 7, a groove was observed on the surfaces of BL SBs just after the first removal. This indicates that a surface change occurred after the first connection. We also observed that the images after 10 connections/disconnections and autoclave treatments did not show apparent differences. This result indicates that the autoclave treatment did not severely affect the surface texture or, possibly, the dimensions of the SBs.

Many studies have been undertaken regarding the accuracy of digital impression techniques by IOS [25,26,27]. The accuracy among these reports ranged from 50 μm to 24 μm, with an inter-implant distance of about 23 mm, whereas the distance between the implant and the analog was 11 mm in the present study. The mean inter-implant distance after autoclave treatment in the present study was close to this range. Thus, there may have been a clinically acceptable level of error after the autoclave treatment. Further studies are needed to determine the required clinical threshold of accuracy.

## 5. Conclusions

In this study, autoclave treatment had some effect on the deformation of the SBs. On the other hand, repeated connection and disconnection of the SBs under the torque of 15 Ncm did not show significant difference. The combined effect of repeated autoclave and connection-disconnection did not show significant difference. From the point of clinical application, the reuse of PEEK SB can be considered under proper sterilization protocol.

## Data Availability

The primary data that support the results described here are available from the corresponding author upon reasonable request.
